# Supplementary material for: Prevention of Testicular Damage by Indole Derivative MMINA via Upregulated StAR and CatSper Channels with Coincident Suppression of Oxidative Stress and Inflammation: In Silico and In Vivo Validation
Source: Antioxidants (Basel). 2022 Oct 19;11(10):2063. doi: 10.3390/antiox11102063 (PMC9598787; doi:10.3390/antiox11102063)
Supplement: Supplementary file 1 [file antioxidants-11-02063-s001.zip › Supplementary File S1.pdf]

Supplementary File: S1

Title: Prevention of testicular damage by indole derivative MMINA via upregulated StAR and CatSper channels with coincident suppression of oxidative stress and inflammation: *In silico* and *in Vivo* validation

Tayyaba Afsar[1], Suhail Razak[1*], Janeen H. Trembley[2,3,4,], Khushbukhat Khan[5,] Maria Shabbir[5,] Ali Almajwal[1,] Nawaf W. Alruwaili[1,] Muhammad Umar Ijaz[5]

Supplementary: Biological Characterization of MMINA

Synthesis of 2-(6-Methoxy-2-methyl-1H-indol-3-yl) acetohydrazide **(1)**

The methyl ester of indomethacin (0.01 mol) and hydrazine hydrate (99%) (0.2 mol) in presence of absolute ethanol (50 mL) were refluxed for 30 hours. The reaction mixture was concentrated by using rota vapor and poured in a beaker containing crushed ice in small portions while stirring and kept for 4 hours at room temperature. The solid was separated out by filtration. The product was dried and recrystallized from ethanol. The product was carefully checked by thin layer chromatography. The first compound was 2-(6-methoxy-2-methyl-1H-indol-3-yl) acetohydrazide compound **(1)**, and was obtained as the major product. The second compound, 4-chlorobenzohydrazide **(2)** was obtained as minor product. Both the compounds were fully characterized by the spectral data.

2-(6-Methoxy-2-methyl-1*H*-indol-3-yl) acetohydrazide (**1**). Color: white; Yield: 70%; m.p.: 168–170 °C; UV λmax (Methanol) = 280 nm; ^{1}H NMR (500 MHz, DMSO-d$_6$): δ = 2.38 (3H, s, CH_3), 3.54 (2H, s, CH_2), 3.80 (3H, s, OCH_3), 4.26 (2H, s, NH_2, D_2O exchg.), 6.67 (1H, d, J = 8.5 Hz, Ar-H), 7.16 (2H, d, J = 7.5 Hz, Ar-H), 9.16 (1H, s, NH, D_2O exchg.), 10.62 (1H, s, CONH, D_2O exchg.); ^{13}C NMR (125 MHz, DMSO-d$_6$): δ = 12.0 (CH_3), 30.2 (CH_2), 55.8 (OCH_3), 101.1, 105.1, 109.8, 110.0, 111.7, 128.0, 129.3, 129.7, 130.6, 134.3, 153.4, 170.8 (C=O); MS: m/z = 233.11 $[M]^+$, 234.07 $[M+1]^+$; Analysis: $C_{12}H_{15}N_3O_2$ for, calcd. C 61.79, H 6.48, N 18.01 %; found C 61.58, H 6.46, N 18.05 %.

4-Chlorobenzohydrazide **(2)**. Color: white; Yield: 20%; M.p.: 148–150 °C; UV λmax (Methanol) = 230 nm; ^{1}H NMR (500 MHz, DMSO-d$_6$): δ = 4.53 (2H, s, NH_2, D_2O exchg.), 7.52 (2H, d, J = 8.5 Hz, Ar-H), 7.84 (2H, d, J = 8.5 Hz, Ar-H), 9.87 (1H, s, CONH, D_2O exchg.); ^{13}C NMR (125 MHz, DMSO-d$_6$): δ = 128.86, 129.32, 132.50, 136.25, 165.29; MS: m/z = 170.45 $[M]^+$; Analysis: $C_7H_7N_2OCl$ for, calcd. C 49.28, H 4.14, N 16.42%; found C 49.37, H 4.12, N 16.46%.

3.3. *General procedure for the synthesis of 2-(5-methoxy-2-methyl-1-indol-3yl)-N-[(E)-substituted phenyl methylidine] aceto hydrazide derivatives* **(S1-S18)**. A solution of indole hydrazide **(1)** (371 mg, 1.0 mmol) in EtOH (15 mL) containing an appropriate substituted benzaldehyde (1.1 mmol) and a catalytic amount of Glacial acetic acid was heated under reflux for 3 hours. After cooling, 5 mL of water was added to the mixture and kept in a refrigerator for 12 hours. The product was obtained by filtration. The compound was washed several times with cold water. The compound obtained was recrystallized from ethanol.

2-(5-Methoxy-2-methyl-1H-indol-3-yl)-N′-[(E)-phenylmethylidene] acetohydrazide **(S1):** Yield: 70%; m.p.: 170–172 °C; IR (KBr) cm^{-1}: 3412 (NH), 3024 (C-H), 1654 (C=O), 1637 (C=N); ^{1}H NMR (500 MHz, DMSO-d$_6$): δ = 2.37 (3H, s, -CH_3), 3.55 (2H, s, CH_2), 3.74 (3H, s, -OCH_3), 6.65-8.00 (8H, m, Ar-H), 10.62 (1H, s, =CH), 11.26 (1H, s, -NH, D_2O exchg.), 11.9 (1H, s, CONH, D_2O exchg.); ^{13}C NMR (125 MHz, DMSO-d$_6$): δ = 12.2 (CH_3), 28.2 (CH_2), 55.5 (OCH_3), 100.7, 104.7, 110.0, 111.3, 127.2, 127.4, 127.6, 129.0, 129.2, 129.3, 130.0, 134.3, 134.8,

153.3, 167.7 (C=O); MS: *m/z* = 321.37 $[M]^+$; Analysis: for $C_{19}H_{19}N_3O_2$, calcd. C 71.01, H 5.96, N 13.08 %; found C 71.25, H 5.94, N 13.11%.

2-(5-Methoxy-2-methyl-1H-indol-3-yl)-N′-[(E)-(4-nitrophenyl)methylidene] acetohydrazide **(S2):** Yield: 75%; m.p.: 220–222 °C; IR (KBr) cm^{-1}: 3411 (NH), 3000 (C-H), 1654 (C=O), 1617 (C=N); ^1H NMR (500 MHz, DMSO-d_6): δ = 2.37 (3H, s, $-CH_3$), 3.58 (2H, s, CH_2), 3.74 (3H, s, $-OCH_3$), 6.97-8.26 (7H, m, Ar-H), 10.63 (1H, s, =CH), 11.70 (1H, s, -NH, D_2O exchg.), 12.2 (1H, s, -CONH, D_2O exchg.); ^{13}C NMR (125 MHz, DMSO-d_6): δ = 11.6 (CH_3), 28.2 (CH_2), 55.0 (OCH_3), 100.2, 110.8, 123.9, 124.0, 127.6, 128.0, 128.6, 129.6, 140.4, 142.0, 143.0, 144.0, 145.5, 151.0, 163.0, 175.0; MS: *m/z* = 366.37 $[M]^+$; Analysis: for $C_{19}H_{18}N_4O_4$, calcd. C 62.29, H 4.95, N 15.29 %; found C 62.14, H 4.97, N 15.25 %.

2-(5-Methoxy-2-methyl-1H-indol-3-yl)-N′-[(E)-(3-nitrophenyl)methylidene] acetohydrazide **(S3):** Yield: 68%; m.p.: 200–202°C; IR (KBr) cm^{-1}: 3412 (NH), 3237 (C-H), 1654 (C=O), 1617 (C=N); ^1H NMR (500 MHz, DMSO-d_6): δ = 2.37 (3H, s, $-CH_3$), 3.58 (2H, s, CH_2), 3.74 (3H, s, $-OCH_3$), 6.99-8.57 (7H, m, Ar-H), 10.63 (1H, s, =CH), 11.5 (1H, s, NH, D_2O exchg.), 12.18 (1H, s, -CONH, D_2O exchg.); ^{13}C NMR (125 MHz, DMSO-d_6): δ = 11.5 (CH_3), 27.9 (CH_2), 55.0, 100.2, 103.9, 109.4, 123.8, 124.2, 128.5, 130.3, 131.7, 133.3, 136.0, 140.33, 145.5, 148.1, 152.8, 162.2, 170.0; MS: *m/z* = 366.37 [M]+; Analysis: for $C_{19}H_{18}N_4O_4$, calcd. C 62.29, H 4.95, N 15.29 %; found C 62.36, H 4.93, N 15.24 %.

2-(5-Methoxy-2-methyl-1H-indol-3-yl)-N′-[(E)-(2-nitrophenyl)methylidene] acetohydrazide **(S4):** Yield: 70%; m.p.: 210–212°C; IR (KBr) cm^{-1}: 3407 (NH), 3063 (C-H), 1654 (C=O), 1617 (C=N); ^1H NMR (500 MHz, DMSO-d_6): δ = 2.37 (3H, s, $-CH_3$), 3.58 (2H, s, CH_2), 3.74 (3H, s, $-OCH_3$), 6.98-8.25 (7H, m, Ar-H), 10.63 (1H, s, =CH), 11.90 (1H, s, -NH, D_2O exchg.), 12.10 (1H, s, -CONH, D2O exchg.); ^{13}C NMR (125 MHz, DMSO-d_6): δ = 11.60 (CH_3), 27.8 (CH_2), 54.9 (OCH_3), 100.0, 103.8, 109.4, 110.6, 124.5, 127.8, 129.6, 133.4, 134.0, 141.4, 143.2, 147.9, 148.2, 152.0; 167.0, 170.0; MS: *m/z* = 366.37 [M]+; Analysis: for $C_{19}H_{18}N_4O_4$, calcd. C 62.29, H 4.95, N 15.29 %; found C 62.15, H 4.97, N 15.24 %

N′-[(E)-(4-chlorophenyl)methylidene]-2-(5-methoxy-2-methyl-1H-indol-3-yl)acetohydrazide **(S5):** Yield: 80%; m.p.: 180–182 °C; IR (KBr) cm^{-1}: 3411 (NH), 3071 (C-H), 1654 (C=O), 1597 (C=N); ^1H NMR (500 MHz, DMSO-d_6): δ = 2.36 (3H, s, $-CH_3$), 3.55 (2H, s, CH_2), 3.74 (3H, s, $-OCH_3$), 6.60-8.45 (7H, m, Ar-H), 10.63 (1H, s, =CH), 11.3 (1H, s, NH, D_2O exchg.), 12.00 (1H, s, -CONH, D_2O exchg.); ^{13}C NMR (125 MHz, DMSO-d_6): δ = 11.6 (CH_3), 28.2 (CH_2), 55.1 (OCH_3), 101.0, 128.5, 128.7, 128.8, 128.9, 129.5, 136.6, 146.7, 148.0, 149.0, 150.0, 151.0, 152.1,162.7, 172.0; MS: *m/z* = 355.81 $[M]^+$; Analysis: for $C_{19}H_{18}N_3O_2Cl$, calcd. C 64.13, H 5.10, N 11.81 %; found C 64.33, H 5.12, N 11.83 %.

N′-[(E)-(2,4-dichlorophenyl)methylidene]-2-(5-methoxy-2-methyl-1H-indol-3-yl)acetohydrazide **(S6)**: Yield: 65%; m.p.: 238–240 °C; IR (KBr) cm^{-1}: 3411 (NH), 2940 (C-H), 1654 (C=O), 1617 (C=N); ^1H NMR (500 MHz, DMSO-d_6): δ = 2.36 (3H, s, $-CH_3$), 3.59 (2H, s, CH_2), 3.74 (3H, s, $-OCH_3$), 6.59-8.61 (6H, m, Ar-H), 10.62 (1H, s, =CH), 11.51 (1H, s, -NH, D_2O exchg.); 11.7 (1H, s, -CONH, D_2O exchg.); ^{13}C NMR (125 MHz, DMSO-d_6): δ = 11.5 (CH_3), 27.8 (CH_2), 55.0 (OCH_3), 100.2, 103.7, 109.4, 110.8, 127.8, 128.7, 129.2, 130.0, 133.4, 133.6, 134.0, 137.5, 140.9, 154.0, 168.0, 172.0S; MS: *m/z* = 390.26 $[M]^+$; Analysis: for $C_{19}H_{17}N_3O_2Cl_2$, calcd. C 58.47, H 4.35, N 10.77 %; found C 58.25, H 4.33, N 10.74 %.

2-(5-Methoxy-2-methyl-1H-indol-3-yl)-N′-[(E)-(3,4-dimethoxyphenyl)methylidene] acetohydrazide **(S7):** Yield: 70%; m.p.: 210–212°C; IR (KBr) cm^{-1}: 3299 (NH), 3011 (C-H), 1654 (C=O), 1599 (C=N); ^1H NMR (500 MHz, DMSO-d_6): δ = 2.40 (3H, s, $-CH_3$), 3.70 (2H, s, CH_2), 3.84 (3H, s, $-OCH_3$), 6.50-8.40 (6H, m, Ar-H), 10.50 (1H, s, =CH), 11.20 (1H, s, -NH, D_2O exchg.), 11.5 (1H, s, -CONH, D_2O exchg.); ^{13}C NMR (125 MHz, DMSO-d_6): δ = 12.1 (CH_3), 12.2 (CH_2), 55.6 (OCH_3), 55.8 (OCH_3), 56.0 (OCH_3), 101.0, 121.4, 122.0, 127.5, 143.1, 151.0, 175.0; MS: *m/z* = 381.42 $[M]^+$; Analysis: for $C_{21}H_{23}N_3O_4$, calcd. C 66.13, H 6.08, N 11.02 %; found C 66.31, H 6.10, N 11.05 %.

2-(5-Methoxy-2-methyl-1H-indol-3-yl)-N′-[(E)-(2-methoxyphenyl)methylidene] acetohydrazide **(S8)**: Yield: 60%; m.p.: 220–222°C; IR (KBr) cm^{-1}: 3315 (NH), 3017 (C-H), 1664 (C=O), 1601 (C=N); ^1H NMR (500 MHz, DMSO-d_6): δ = 2.36 (3H, s, $-CH_3$), 3.56 (2H, s, CH_2), 3.87 (3H, s, $-OCH_3$), 7.00-8.82 (11H, m, Ar-H), 10.61 (1H, s, =CH), 11.23(1H, s, NH, D_2O exchg.), 11.92 (1H, s, -CONH, D_2O exchg.); ^{13}C NMR (125 MHz, DMSO-d_6): δ = 12.1

(CH_3), 28.1 (CH_2), 56.1 (OCH_3), 63.4 (OCH_3), 111.2, 112.2, 112.3, 121.2, 122.7, 125.8, 126.0, 129.0, 130.0, 132.1, 137.0, 144.0, 158.2, 162.3; MS: m/z = 351.39 $[M]^+$; Analysis: for $C_{20}H_{21}N_3O_3$, calcd. C 68.36, H 6.02, N 11.96 %; found C 68.50, H 6.00, N 11.93 %.

N′-[(E)-(4-hydroxyphenyl)methylidene]-2-(5-methoxy-2-methyl-1H-indol-3-yl)acetohydrazide (**S9):** Yield: 70%; m.p.: 230–232 °C; IR (KBr) cm^{-1}: 3411 (OH), 3411 (NH), 3300 (C-H), 1654 (C=O), 1609 (C=N); ^1H NMR (500 MHz, DMSO-d_6): δ = 2.37 (3H, s, -CH_3), 3.58 (2H, s, CH_2), 3.75 (3H, s, -OCH_3), 6.59-8.36 (7H, m, Ar-H), 9.88 (1H, s, OH, D_2O exchg.), 10.60 (1H, s, =CH), 11.0 (1H, s, -CONH, D_2O exchg.), 11.73 (1H, s, -CONH, D_2O exchg.); ^{13}C NMR (125 MHz, DMSO-d_6): δ = 12.1 (CH_3), 28.2 (CH_2), 55.5 (OCH_3), 100.8, 104.9, 109.8, 110.0, 116.1, 128.9, 129.4, 130.5, 134.2, 143.4, 153.3, 159.5, 162.2, 167.4, 170.7, 172.9; MS: m/z = 337.37 $[M]^+$; Analysis: for $C_{19}H_{19}N_3O_3$, calcd. C 67.64, H 5.68, N 12.46 %; found C 67.43, H 5.70, N 12.43 %.

N′-[(E)-(3-hydroxyphenyl)methylidene]-2-(5-methoxy-2-methyl-1H-indol-3-yl)acetohydrazide (**S10**): Yield: 60%; m.p.: 145–147 °C; IR (KBr) cm^{-1}: 3500 (OH), 3413 (NH), 3023 (C-H), 1654 (C=O), 1617 (C=N); ^1H NMR (500 MHz, DMSO-d_6): δ = 2.37 (3H, s, -CH_3), 3.57 (2H, s, CH_2), 3.74 (3H, s, -OCH_3), 6.58-8.17 (7H, m, Ar-H), 9.59 (1H, s, OH, D_2O exchg.), 10.62 (1H, s, =CH), 11.21 (1H, s, -NH, D_2O exchg.), 11.39 (1H, s, -CONH, D_2O exchg.); ^{13}C NMR (125 MHz, DMSO-d_6): δ = 11.6 (CH_3), 27.6 (CH_2), 54.9 (OCH_3), 100.1, 104.0, 109.3, 110.8, 112.4, 117.2, 118.2, 128.5, 129.5, 130.0, 133.8, 135.5, 146.2, 152.8, 157.5, 175.0; MS: m/z = 337.37 [M]+; Analysis: for $C_{19}H_{19}N_3O_3$, calcd. C 67.64, H 5.68, N 12.46 %; found C 67.41, H 5.70, N 12.42 %.

2-(5-Methoxy-2-methyl-1H-indol-3-yl)-N′-{(E)-[4-(dimethylamino) phenyl]methylidene}acetohydrazide (**S11**): Yield: 65%; m.p.: 200–202 °C; IR (KBr) cm^{-1}: 3351 (NH), 2909 (C-H), 1654 (C=O), 1609 (C=N); ^1H NMR (500 MHz, DMSO-d_6): δ = 2.37 (3H, s, -CH_3), 3.00 (6H, s, 2×NCH_3) 3.59 (2H, s, CH_2), 3.74 (3H, s, -OCH_3), 6.59-8.32 (7H, m, Ar-H), 10.60 (1H, s, =CH), 10.97 (1H, s, -NH, D_2O exchg.), 11.62 (1H, s, -CONH, D_2O exchg.); ^{13}C NMR (125 MHz, DMSO-d_6): δ = 12.1 (CH_3), 28.0 (CH_2), 30.0 (NCH_3), 31.0 (NCH_3), 55.5 (OCH_3), 100.9, 105.0, 110.0, 111.2, 112.27, 112.3, 122.2, 128.4, 128.7, 128.9, 129.8, 130.5, 143.9, 151.7, 153.3, 172.7; ms: m/z = 364.44 $[M]^+$; Analysis: for $C_{21}H_{24}N_4O_2$, calcd. C 69.21, H 6.64, N 15.37 %; found C 69.37, H 6.66, N 15.33 %.

2-(5-methoxy-2-methyl-1H-indol-3-yl)-N′-[(E)-(3-methoxyphenyl)methylidene] acetohydrazide (**S12**): Yield: 65%; m.p.: 195–197 °C; IR (KBr) cm^{-1}: 3412 (NH), 3000 (C-H), 1654 (C=O), 1636 (C=N); ^1H NMR (500 MHz, DMSO-d_6): δ = 2.37 (3H, s, -CH_3), 3.58 (2H, s, CH_2), 3.80 (3H, s, -OCH_3), 6.59-8.44 (7H, m, Ar-H), 10.67 (1H, s, =CH), 11.28 (1H, s, -NH, D_2O exchg.), 11.46 (1H, s, -CONH, D_2O exchg.); ^{13}C NMR (125 MHz, DMSO-d_6): δ = 12.1 (CH_3), 28.3 (CH_2), 55.5 (OCH_3), 55.35 (OCH_3), 100.8, 104.5, 104.7, 109.8, 110.0, 111.6, 120.3, 129.3, 130.3, 134.3, 134.3, 136.2, 142.9, 146.5, 153.3, 159.9, 173.3; MS: m/z = 351.39 $[M]^+$; Analysis: for $C_{20}H_{21}N_3O_3$, calcd. C 68.36, H 6.02, N 11.96 %; found C 68.15, H 6.00, N 11.99 %.

N′-[(E)-(4-ethoxyphenyl)methylidene]-2-(5-methoxy-2-methyl-1H-indol-3-yl)acetohydrazide (**S13**): Yield: 75%; m.p.: 213–215 °C; IR (KBr) cm^{-1}: 3322 (NH), 3042 (C-H), 1654 (C=O), 1571 (C=N); ^1H NMR (500 MHz, DMSO-d_6): δ = 1.33 (3H, t, J = 7.5 Hz, CH_2CH_3), 2.37 (3H, s, -CH_3), 3.57 (2H, s, CH_2), 3.74 (3H, s, -OCH_3), 4.06 (2H, q, J = 7.5 Hz, OCH_2), 6.58-8.20 (7H, m, Ar-H), 10.61 (1H, s, =CH), 11.12 (1H, s, -NH, D_2O exchg.), 11.30 (1H, s, -CONH, D_2O exchg.); ^{13}C NMR (125 MHz, DMSO-d_6): δ = 12.2 (CH_3), 15.0 (CH_2), 28.2 (CH_3), 55.5 (OCH_3), 55.88 (OCH_2), 63.7, 100.8, 104.8, 109.8, 111.2, 115.1, 127.2, 129.2, 129.3, 130.5, 134.3, 143.0, 146.5, 153.3, 160.2, 167.5, 173.0; ms: m/z = 365.42 $[M]^+$; Analysis: for $C_{21}H_{23}N_3O_3$, calcd. C 69.02, H 6.34, N 11.50 %; found C 69.22, H 6.36, N 11.53 %.

2-(5-Methoxy-2-methyl-1H-indol-3-yl)-N′-[(E)-(2,4,5-trimethoxyphenyl) methylidene]acetohydrazide (**S14**): Yield: 60%; m.p.: 238–240 °C; IR (KBr) cm^{-1}: 3412 (NH), 2943 (C-H), 1654 (C=O), 1617 (C=N); ^1H NMR (500 MHz, DMSO-d_6): δ = 2.36 (3H, s, -CH_3), 3.51 (2H, s, CH_2), 3.78 (12H, s, -4×OCH_3), 6.91-8.42 (5H, m, Ar-H), 10.61 (1H, s, =CH), 11.14 (1H, s, -NH, D_2O exchg.), 11.42 (1H, s, -CONH, D_2O exchg.); ^{13}C NMR (125 MHz, DMSO-d_6): δ = 12.1 (CH_3), 30.1 (CH_2), 55.5 (OCH_3), 56.4 (OCH_3), 60.9 (OCH_3), 62.1 (OCH_3), 100.8, 104.8, 109.1, 109.8, 110.0, 111.2, 120.8, 130.5, 134.4, 138.9, 142.0, 152.8, 153.3, 155.2, 172.9; ms: m/z = 411.45 $[M]^+$; Analysis: for $C_{22}H_{25}N_3O_5$, calcd. C 64.22, H 6.12, N 10.21 %; found C 64.35, H 6.14, N 10.24 %.

2-(5-Methoxy-2-methyl-1H-indol-3-yl)-N′-[(E)-(2,3,4-trimethoxyphenyl) methylidene]acetohydrazide (**S15**): Yield: 55%; m.p.: 250–252 °C; IR (KBr) cm^{-1}: 3310 (NH), 3048 (C-H), 1654 (C=O), 1595 (C=N); ^1H NMR (500 MHz,

DMSO-d_6): δ = 2.37 (3H, s, -CH_3), 3.59 (2H, s, CH_2), 3.84 (12H, s, 4×-OCH_3), 6.59-8.74 (5H, m, Ar-H), 10.60 (1H, s, =CH), 11.08 (1H, s, -NH, D_2O exchg.), 11.33 (1H, s, -CONH, D_2O exchg.); ^{13}C NMR (125 MHz, DMSO-d_6): δ = 12.1 (CH_3), 28.5 (CH_2), 55.6 (OCH_3), 56.2 (OCH_3), 56.4 (OCH_3), 56.9 (OCH_3), 98.3, 101.1, 104.8, 108.4, 109.8, 111.2, 114.1, 129.2, 130.6, 134.4, 138.8, 143.6, 152.1, 153.6, 167.3, 172.9; ms: *m/z* = 411.45 [M]+; Analysis: for $C_{22}H_{25}N_3O_5$, calcd. C 64.22, H 6.12, N 10.21 %; found C 64.36, H 6.10, N 10.23 %.

2-(5-Methoxy-2-methyl-1H-indol-3-yl)-N′-[(E)-(3,4,5-trimethoxyphenyl) methylidene]acetohydrazide (**S16**): Yield: 58%; m.p.: 233–235 °C; IR (KBr) cm^{-1}: 3309 (NH), 3015 (C-H), 1654 (C=O), 1577 (C=N); ^1H NMR (500 MHz, DMSO-d_6): δ = 2.37 (3H, s, -CH_3), 3.59 (2H, s, CH_2), 3.83 (12H, s, 4× -OCH_3), 6.97-8.20 (5H, m, Ar-H), 10.61 (1H, s, =CH), 11.28 (1H, s, -NH, D_2O exchg.), 11.40 (1H, s, -CONH, D_2O exchg.); ^{13}C NMR (125 MHz, DMSO-d_6): δ = 12.1 (CH_3), 28.4 (CH_2), 55.6 (OCH_3), 55.8 (OCH_3), 56.3 (OCH_3), 60.5 (OCH_3), 60.5, 101.1, 104.4, 104.6, 104.7, 109.8, 111.2, 130.3, 130.6, 134.2, 139.3, 142.9, 153.3, 153.6, 173.2; ms: *m/z* = 411.45 [M]+; Analysis: for $C_{22}H_{25}N_3O_5$, calcd. C 64.22, H 6.12, N 10.21 %; found C 64.37, H 6.10, N 10.24 %.

2-(5-Methoxy-2-methyl-1H-indol-3-yl)-N′-[(E)-(2,4,6-trimethoxyphenyl) methylidene]acetohydrazide (**S17**): Yield: 55%; m.p.: 230–232 °C; IR (KBr) cm^{-1}: 3412 (NH), 3056 (C-H), 1654 (C=O), 1612 (C=N); ^1H NMR (500 MHz, DMSO-d_6): δ = 2.37 (3H, s, -CH_3), 3.59 (2H, s, CH_2), 3.82 (12H, s, 4× -OCH_3), 6.57-8.74 (5H, m, Ar-H), 10.61 (1H, s, =CH), 11.10 (1H, s, NH, D_2O exchg.), 11.80 (1H, s, -CONH, D_2O exchg.); ^{13}C NMR (125 MHz, DMSO-d_6): δ = 12.1 (CH_3), 28.4 (CH_2), 55.5 (OCH_3), 55.7 (OCH_3), 56.3 (OCH_3), 60.5 (OCH_3), 98.6, 106.7, 111.2, 115.6, 129.9, 142.2, 144.1, 153.3, 159.3, 159.6 162.1, 167.3, 172.9; ms: *m/z* = 411.45 [M]+; Analysis: for $C_{22}H_{25}N_3O_5$, calcd. C 64.22, H 6.12, N 10.21 %; found C 64.38, H 6.13, N 10.17 %.

2-(5-Methoxy-2-methyl-1H-indol-3-yl)-N′-[(E)-(2,4-dimethoxyphenyl) methylidene]acetohydrazide (**S18**): Yield: 60%; m.p.: 170–172 °C; IR (KBr) cm^{-1}: 3413 (NH), 3000 (C-H), 1654 (C=O), 1638 (C=N); ^1H NMR (500 MHz, DMSO-d_6): δ = 2.40 (3H, s, -CH_3), 3.60 (2H, s, CH_2), 3.82 (3H, s, -OCH_3), 6.50-8.70 (6H, m, Ar-H), 10.61 (1H, s, =CH), 11.20 (1H, s, NH, D_2O exchg.); 11.80 (1H, s, -CONH, D_2O exchg.); ^{13}C NMR (125 MHz, DMSO-d_6): δ = 11.5 (CH_3), 28.4 (CH_2), 55.0 (OCH_3), 55.6 (OCH_3), 55.68 (OCH_3), 98.2, 104.4, 106.2, 109.5, 110.7, 115.2, 128.8, 130.0, 132.0, 135.8, 138.3, 143.6, 152.8, 158.9, 162.0, 162.4, 164.7, 172.0; ms: *m/z* = 381.42 $[M]^+$; Analysis: for $C_{21}H_{23}N_3O_4$, calcd. C 66.13, H 6.08, N 11.02 %; found C 66.34, H 6.10, N 11.05 %.

Supplementary Table 1: Primers

Candidate gene	primer
**StAR**	F: 5’-GGG CTC CACCTG CTT CTT G-3’<br>R: 5’- ACC CCT CTG CTC AGG CAT TT −3’
**HSD**	F: 5’-GAAGAAGCATGGAGGTCAAC-3’<br>R: 5’-GCAATCAGAGGTTGGGTCAT-3’.′
**CYP20A1**	F: 5′-CCTCGTCCTTTATGCCCTTGGT-3′<br>R: 5′-GTGCCTGAGAATCCAAGTGAGG-3′
**CAT Sper1**	F: 5'-TCGGAGAACCACAGAGAAGAG-3’<br>R: 5'-CACACACCGGGAATATCTTC-3'
**CAT Sper2**	F: 5'-TGGCCACAGAGCAGTATTTG-3′<br>R: 5'-TGTCAGGCTGTTGCTTTGTC-3’
**iNOs**	F: 5′-CTATGGCCGCTTTGATGTGC-3′<br>R: 5′-CAACCTTGGTGTTGAAGGCG-3′

Scheme1

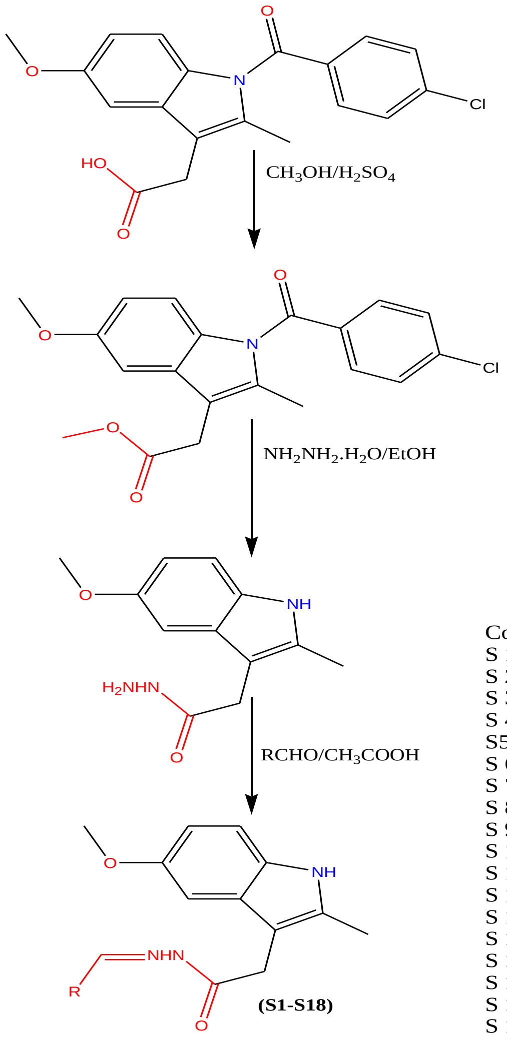

(S1-S18)

Code	R
S 1	Phenyl
S 2	4-Nitrophenyl
S 3	3-Nitrophenyl
S 4	2-Nitrophenyl
S5	4-Chlorophenyl
S 6	2, 4-Dichlorophenyl
S 7	3,4-Dimethoxyphenyl
S 8	2-Methoxyphenyl
S 9	4-Hydroxyphenyl
S 10	3-Hydroxyphenyl
S 11	4- Dimethylaminophenyl
S 12	3-methoxyphenyl
S 13	4-Ethoxyphenyl
S 14	2, 4, 5-Trimethoxyphenyl
S 15	2, 3, 4-Trimethoxyphenyl
S 16	3, 4, 5-Trimethoxyphenyl
S 17	2, 4, 6-Trimethoxyphenyl
S 18	2, 4-Dimethoxyphenyl
