# Supplementary material for: Prevention of Testicular Damage by Indole Derivative MMINA via Upregulated StAR and CatSper Channels with Coincident Suppression of Oxidative Stress and Inflammation: In Silico and In Vivo Validation
Source: Antioxidants (Basel). 2022 Oct 19;11(10):2063. doi: 10.3390/antiox11102063 (PMC9598787; doi:10.3390/antiox11102063)
Supplement: Supplementary file 1 [file antioxidants-11-02063-s001.zip › suplimentary 2.pdf]

Supplementary File: S2

Title: Prevention of testicular damage by indole derivative MMINA via upregulated StAR and CatSper channels with coincident suppression of oxidative stress and inflammation: *In silico* and *in Vivo* validation

Tayyaba Afsar[1], Suhail Razak[1*], Janeen H. Trembley[2,3,4,] , Khushbukhat Khan[5,] Maria Shabbir[5,] Ali Almajwal[1,] Nawaf W. Alruwaili[1,] Muhammad Umar Ijaz[5]

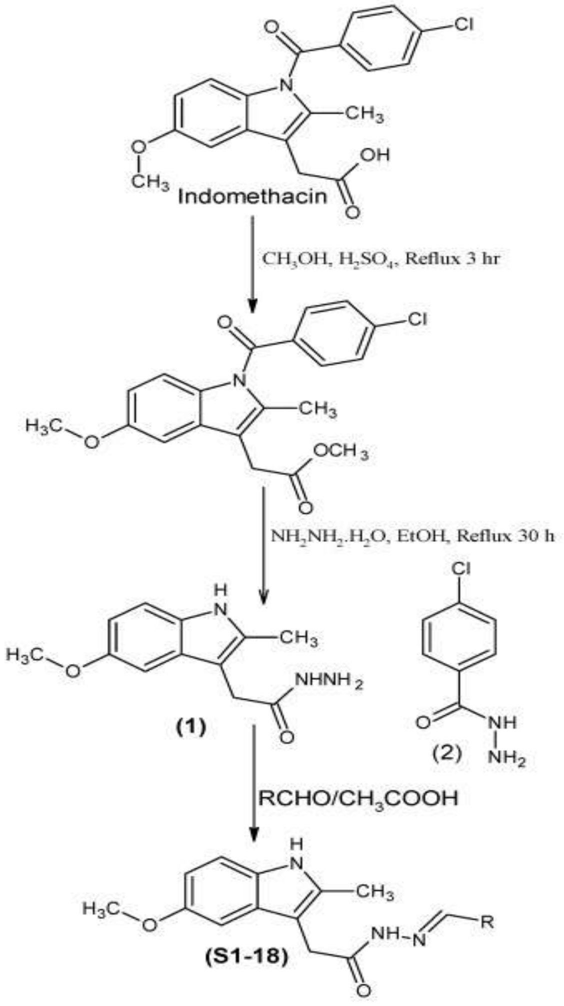

Compound Code	R
S 1	Phenyl
S 2	4-Nitrophenyl
S 3	3-Nitrophenyl
S 4	2-Nitrophenyl
S5	4-Chlorophenyl
S 6	2, 4-Dichlorophenyl
S 7	3,4-Dimethoxyphenyl
S 8	2-Methoxyphenyl
S 9	4-Hydroxyphenyl
S 10	3-Hydroxyphenyl
S 11	4- Dimethylaminophenyl
S 12	3-methoxyphenyl
S 13	4-Ethoxyphenyl
S 14	2, 4, 5-Trimethoxyphenyl
S 15	2, 3, 4-Trimethoxyphenyl
S 16	3, 4, 5-Trimethoxyphenyl
S 17	2, 4, 6-Trimethoxyphenyl
S 18	2, 4-Dimethoxyphenyl

Supplementary file S2, Scheme 1
