# Supplementary material for: Prevention of Testicular Damage by Indole Derivative MMINA via Upregulated StAR and CatSper Channels with Coincident Suppression of Oxidative Stress and Inflammation: In Silico and In Vivo Validation
Source: Antioxidants (Basel). 2022 Oct 19;11(10):2063. doi: 10.3390/antiox11102063 (PMC9598787; doi:10.3390/antiox11102063)

Supplementary File: S3

Title: Prevention of testicular damage by indole derivative MMINA via upregulated StAR and CatSper channels with coincident suppression of oxidative stress and inflammation: *In silico* and *in Vivo* validation

Tayyaba Afsar[1], Suhail Razak[1*], Janeen H. Trembley[2,3,4], Khushbukhat Khan[5], Maria Shabbir[5], Ali Almajwal[1], Nawaf W. Alruwaili[1], Muhammad Umar Ijaz[5]

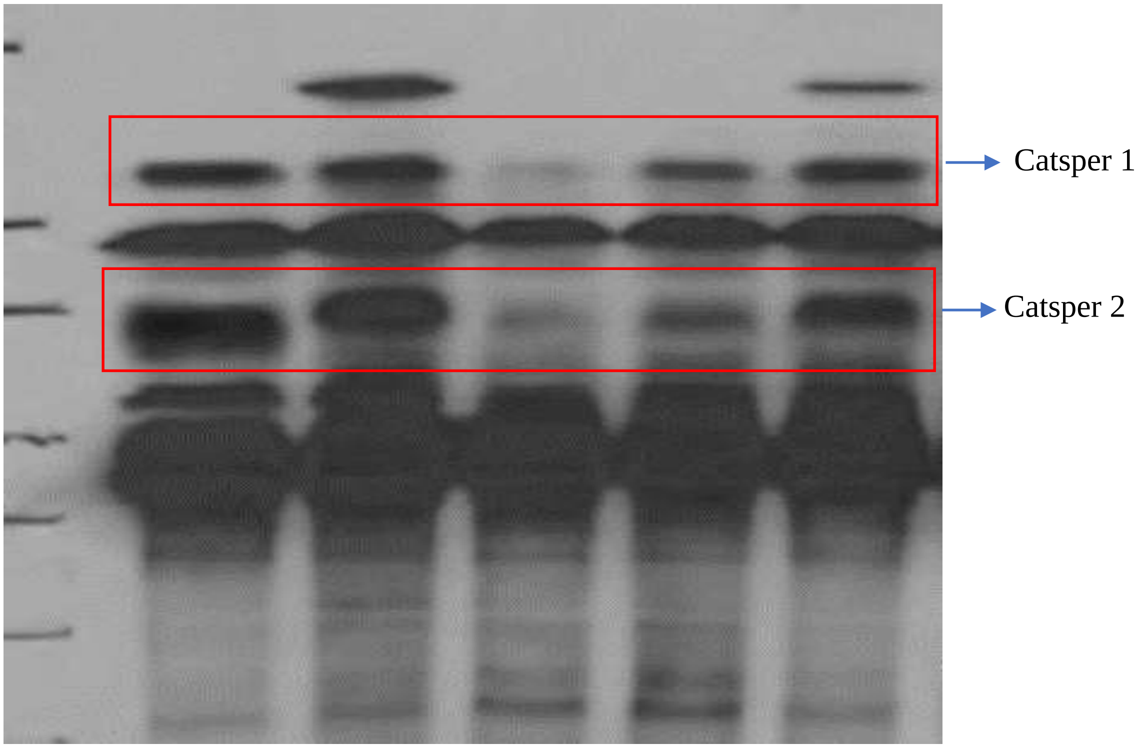

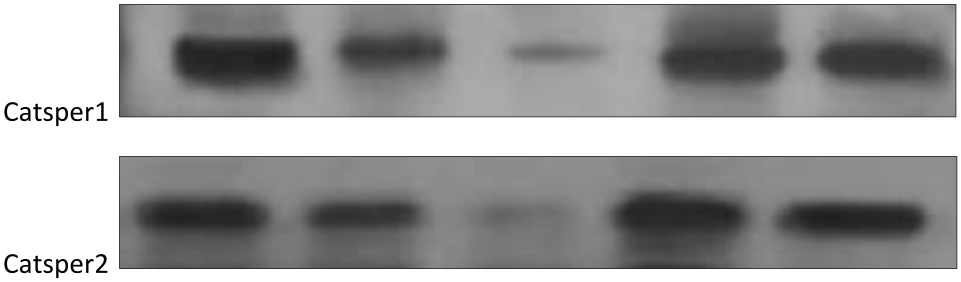

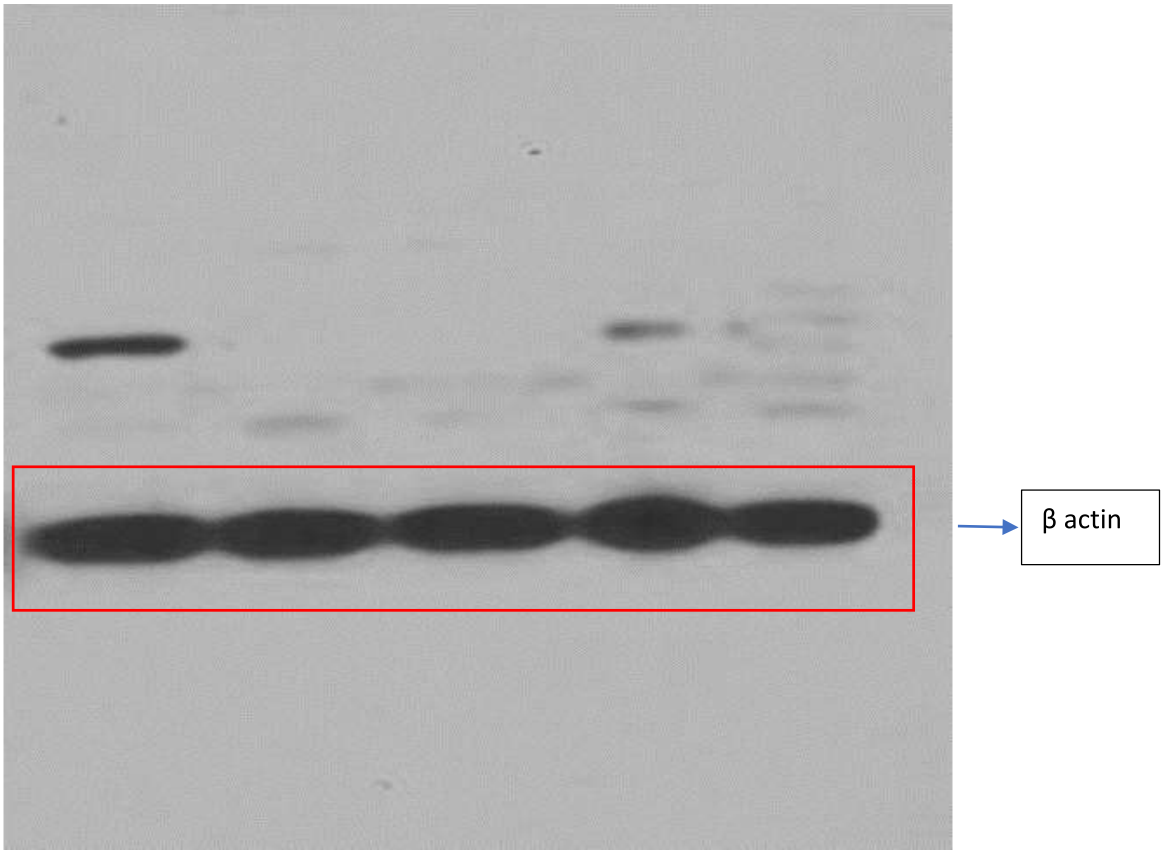

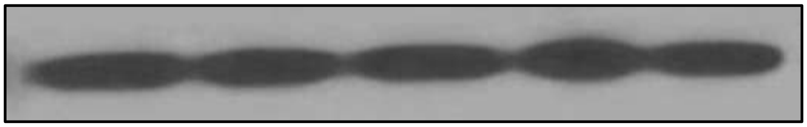

β actin

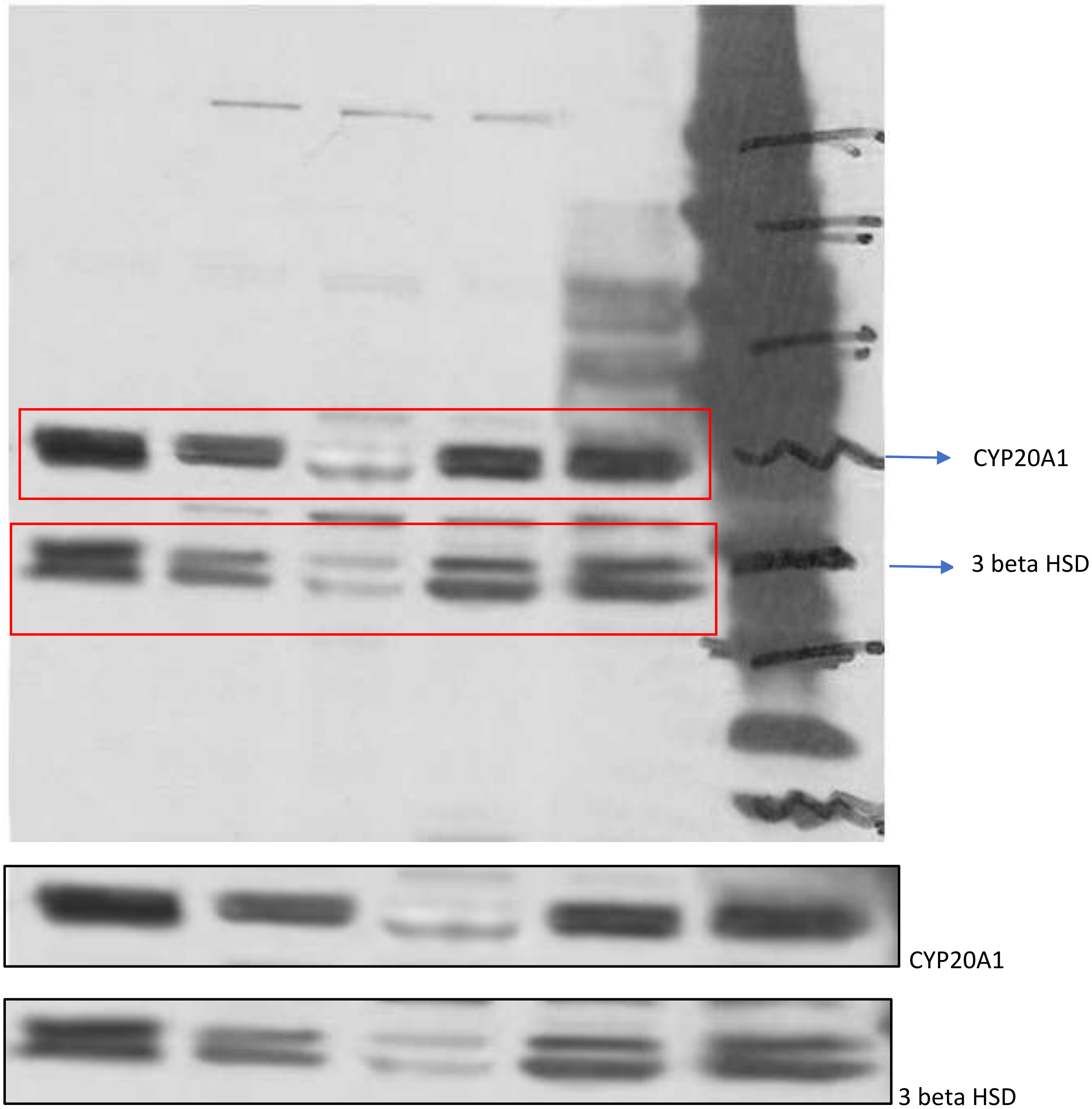
CYP20A1
3 beta HSD
CYP20A1
3 beta HSD

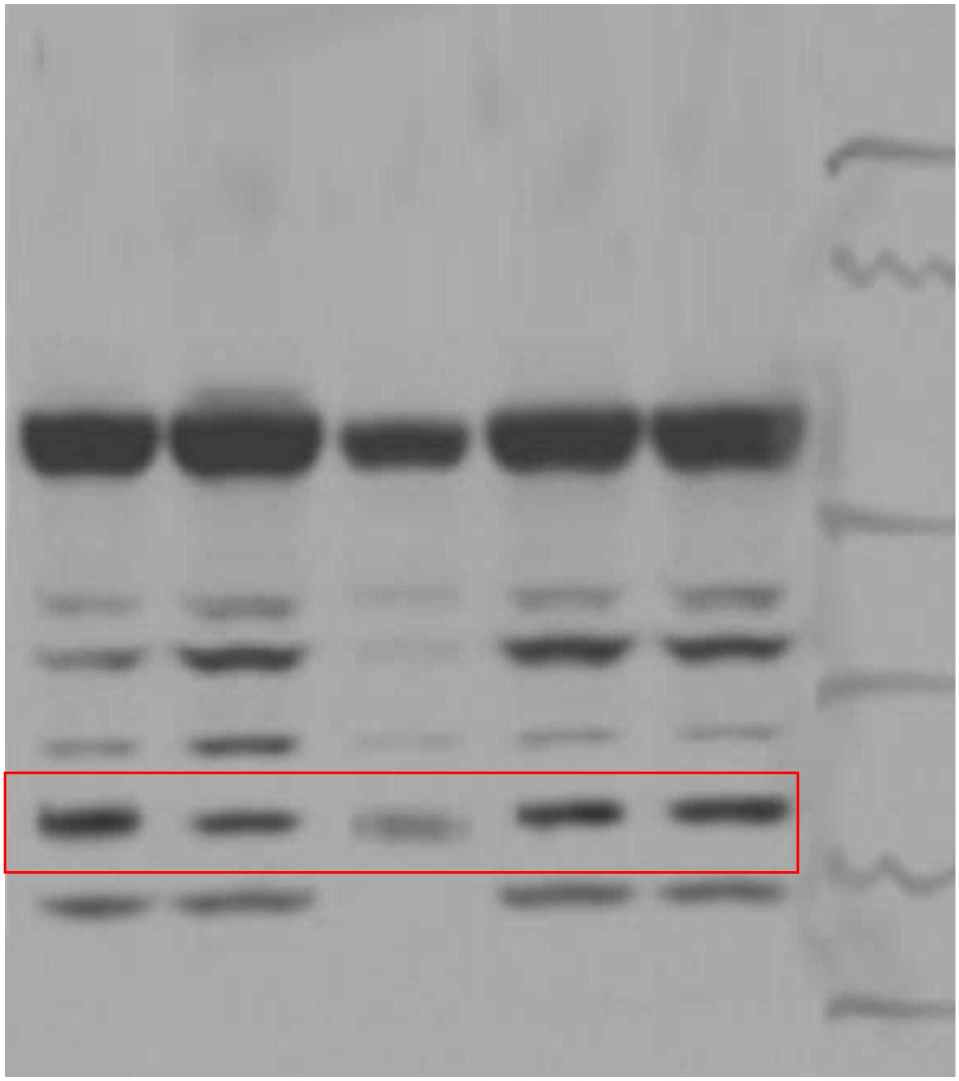

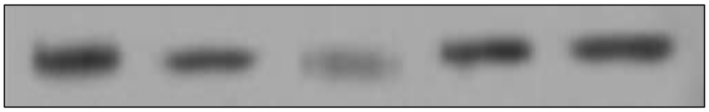

StAR

\

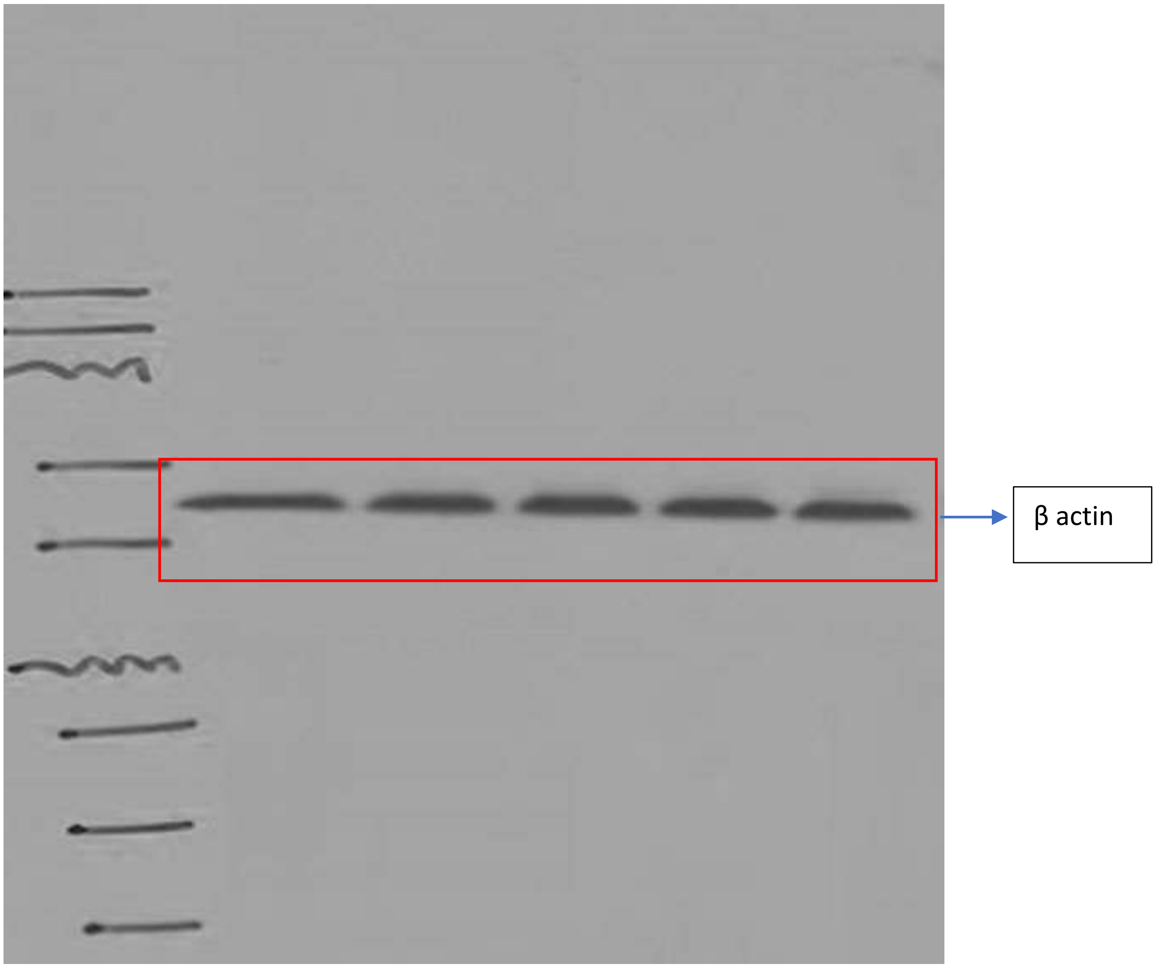

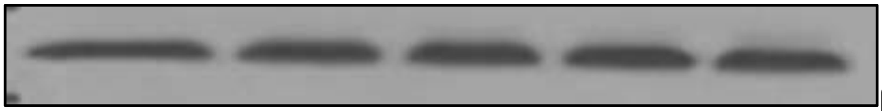
β-actin

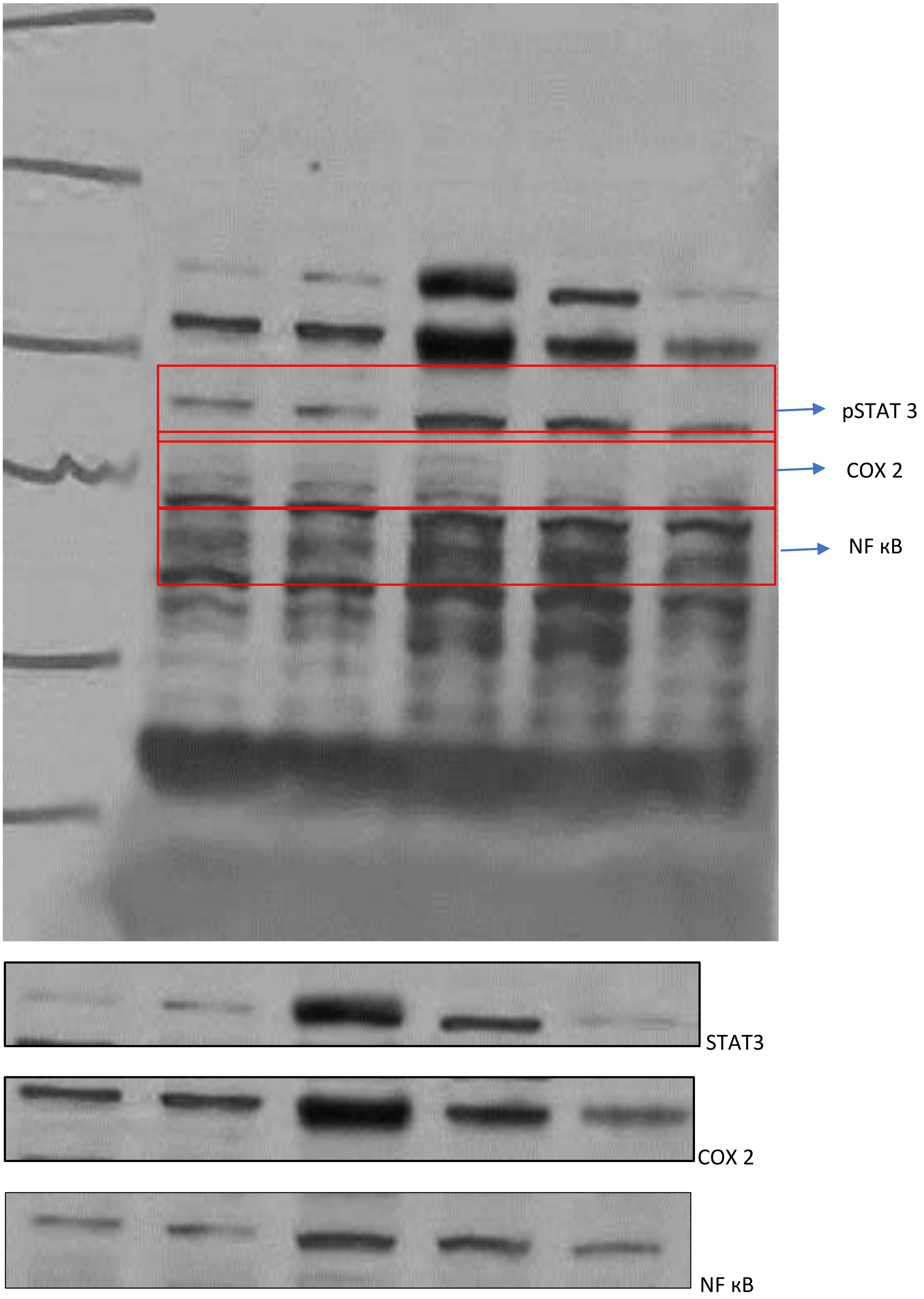
pSTAT 3
COX 2
NF κB
STAT3
COX 2
NF κB

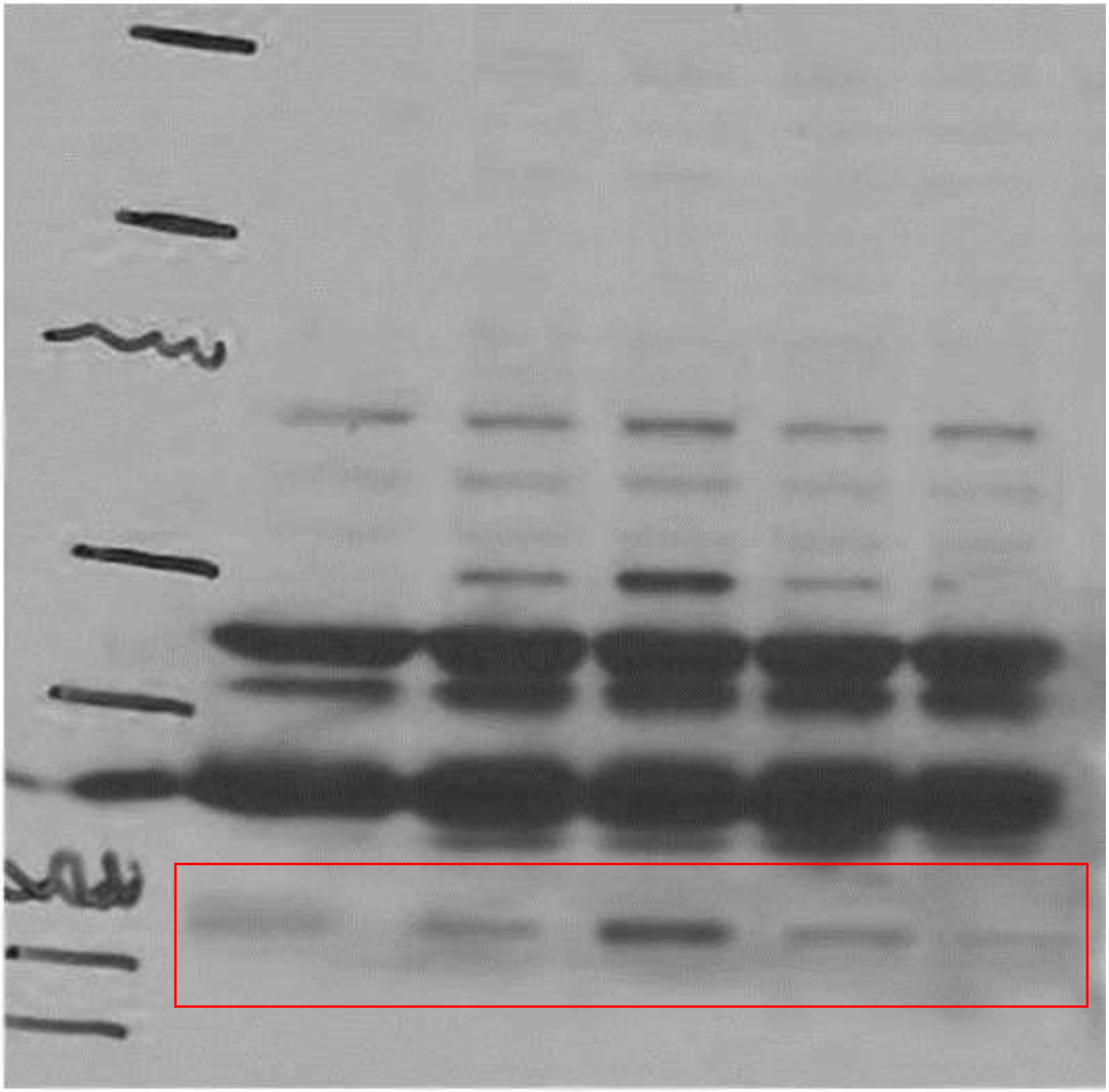

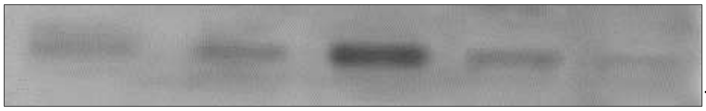
TNF alpha

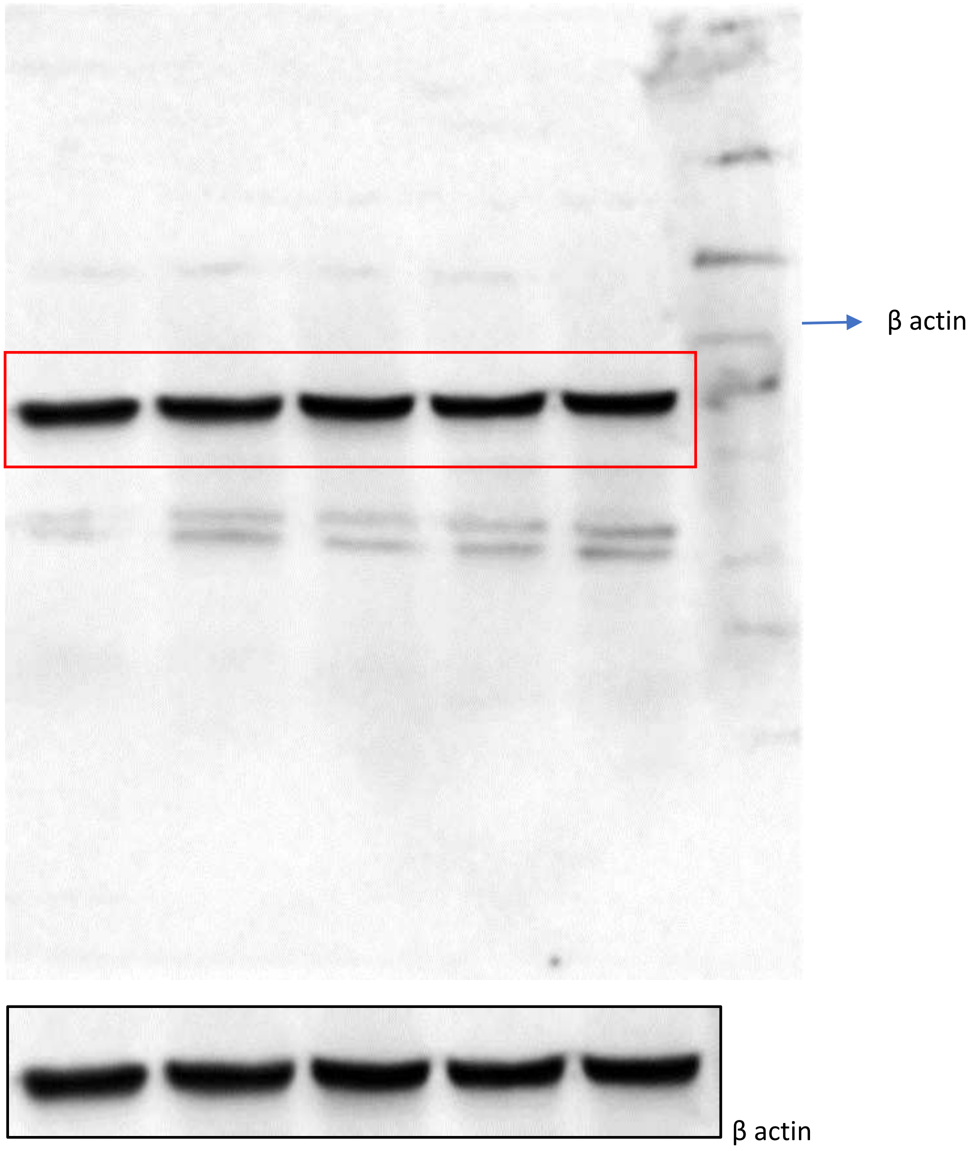
β actin
β actin

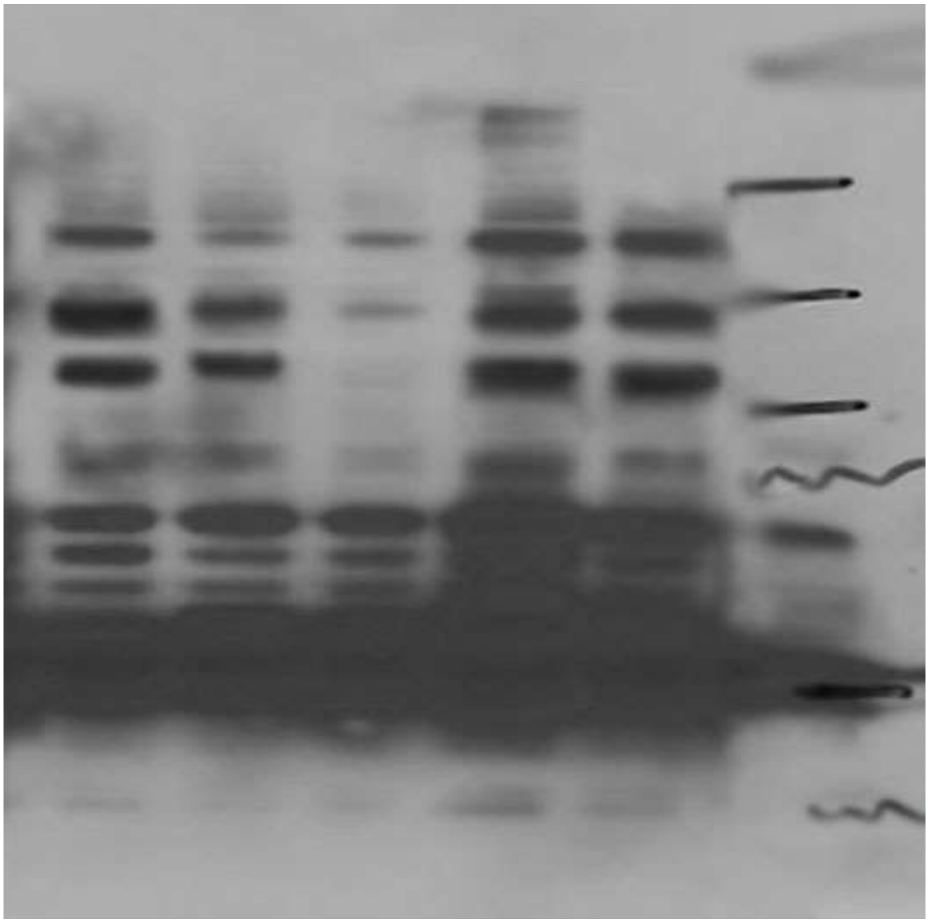

Supplement: Supplementary file 1 [file antioxidants-11-02063-s001.zip › Supplimentary file 3 western bloting.pdf]
