# Supplementary material for: Prevention of Testicular Damage by Indole Derivative MMINA via Upregulated StAR and CatSper Channels with Coincident Suppression of Oxidative Stress and Inflammation: In Silico and In Vivo Validation
Source: Antioxidants (Basel). 2022 Oct 19;11(10):2063. doi: 10.3390/antiox11102063 (PMC9598787; doi:10.3390/antiox11102063)
Supplement: Supplementary file 1 [file antioxidants-11-02063-s001.zip › Supplimentary file 4.docx.pdf]

Supplementary File: S4

Title: Prevention of testicular damage by indole derivative MMINA via upregulated StAR and CatSper channels with coincident suppression of oxidative stress and inflammation: *In silico* and *in Vivo* validation

Tayyaba Afsar[1], Suhail Razak[1*], Janeen H. Trembley[2,3,4,] , Khushbukhat Khan[5,] Maria Shabbir[5,] Ali Almajwal[1,] Nawaf W. Alruwaili[1,] Muhammad Umar Ijaz[5]

Supplementary Table 2: Docked complexes of inflammatory pathway proteins with MMINA along with vina score and cavity size

Drug	Protein	Vina score	Cavity size
MMINA	COX3	-9.1	1312
		-8.9	4932
		-7.4	994
		-7.4	196
		-6.5	224
	TNF alpha	-7.6	303
		-7.1	59
		-7	92
		-6.3	53
		-6.1	66
	NF-kB	-7.3	219
		-6.9	362
		-6.7	305
		-6.5	692
		-4.9	164
	STAT3	-8.6	504
		-7.9	3885
		-7.7	1286
		-7.2	2161
		-5.9	510

Supplementary Table 3: Docked complexes of testicular function proteins with MMINA along with vina score and cavity size

Drug	Protein	Vina score	Cavity size
MMINA	3beta HSD	-9.6	3266

		-7.4	289
		-6.8	264
		-6.6	194
		-5.7	211
	CATSPERZ	-7.7	272
		-7.1	97
		-6.6	101
		-6.6	95
		-6.3	92
	CYP20A1	-7.6	469
		-7.5	14045
		-7.3	215
		-7.1	674
		-7	338
	StAR	-9	1425
		-7.3	112
		-7.2	429
		-7	300
		-6.7	162
